# Robotic Surgery in Rectal Cancer: Potential, Challenges, and Opportunities

**DOI:** 10.1007/s11864-022-00984-y

**Published:** 2022-04-18

**Authors:** Ge Liu, Shoujia Zhang, Yan Zhang, Xiaoqing Fu, Xinlu Liu

**Affiliations:** 1 1st Department of General Surgery, The First Affiliated Hospital of Dalian Medical University, No. 193 Union Road, Dalian City, Liaoning Province People’s Republic of China; 2 Operating Room, The First Affiliated Hospital of Dalian Medical University, No. 193 Union Road, Dalian City, Liaoning Province People’s Republic of China

**Keywords:** Colorectal cancer, Robotic surgery, RTME, RTaTME, RLLND

## Abstract

The current standard treatment for locally advanced rectal cancer is based on a multimodal comprehensive treatment combined with preoperative neoadjuvant chemoradiation and complete surgical resection of the entire mesorectal cancer. For ultra-low cases and cases with lateral lymph node metastasis, due to limitations in laparoscopic technology, the difficulties of operation and incidence of intraoperative complications are always difficult to overcome. Robotic surgery for the treatment of rectal cancer is an emerging technique that can overcome some of the technical drawbacks posed by conventional laparoscopic approaches, improving the scope and effect of radical operations. However, evidence from the literature regarding its oncological safety and clinical outcomes is still lacking. This brief review summarized the current status of robotic technology in rectal cancer therapy from the perspective of several mainstream surgical methods, including robotic total mesorectal excision (TME), robotic transanal TME, robotic lateral lymph node dissection, and artificial intelligence, focusing on the developmental direction of robotic approach in the field of minimally invasive surgery for rectal cancer in the future.

## Introduction

The incidence rate of rectal cancer in colorectal cancer (CRC) is 65% [1]. Surgery in combination with chemoradiation is the standard treatment modality for rectal cancer. Minimally invasive surgery has become the mainstream of modern colorectal surgery worldwide. Several prospective and randomized trials have demonstrated that there is no difference in postoperative prognosis between laparoscopic and open surgery approaches in rectal cancer treatment [2–4]. However, according to the limited range of device mobility in the narrow pelvic cavity, less dexterity, inadequate vision of the operative field, and artificial tremors, laparoscopic rectal cancer surgery is not at the apex of technology; instead, it needs further improvement, innovation, and even substitution.

With the aid of robotic colorectal surgery, rectal cancer treatment has entered a new era of an advanced form of minimally invasive surgery. Since the first successful surgery using the da Vinci Surgical System (Intuitive Surgical Inc., Sunnyvale, CA, USA) in 2000, as many as 1,037,000 procedures have been performed in 67 countries [5]. To date, the da Vinci System (available models: da Vinci Si, X, Xi, SP) is the most widely used robotic surgical system globally. As a new emerging technique, robotic surgery is considered to overcome the shortcomings of laparoscopic surgery and open a new era of minimally invasive surgery. Currently, the mainstream robotic surgery system is integrated with three elements: a surgeon console, patient-side cart with interactive robotic arms connected to the surgical instruments, and video tower with system processors with a high-definition three-dimensional (3D) vision system. The evident advantages of robotic surgical systems are improvement in dexterity, increased range of movements at the tips of the instruments, enhanced ergonomics, elimination of physiologic tremors, and a stable camera with a 3D view. Thus, a robotic system has more remarkable advantages in performing a higher-quality operation in a narrow space (such as the pelvic cavity), compared to conventional laparoscopy.

This paper reviews the application status of robot technology in several mainstream radical resection of rectal cancer from the perspective of surgery, including robotic total mesorectal excision (RTME), robotic transanal TME (RTaTME), and robotic lateral lymph node dissection (RLLND), and future developmental direction with the participation of artificial intelligence (AI). We hope that the summarized information in this review can truly reflect the current state of robotic rectal cancer surgery and shed light on the future of rectal surgery.

## Robotics in total mesorectal excision (TME) surgery in locally advanced rectal cancer

A clear circumferential resection margin (CRM) of rectal cancer is defined as a range of >1 mm from the tumor tissue to the surgical radial margin. As a consensus, TME is crucial for satisfying oncological outcomes. According to the previous study, TME surgery has a lower local recurrence (LR) rate (<10%) compared to previous conventional dissection [6].

**Fig. 1.**
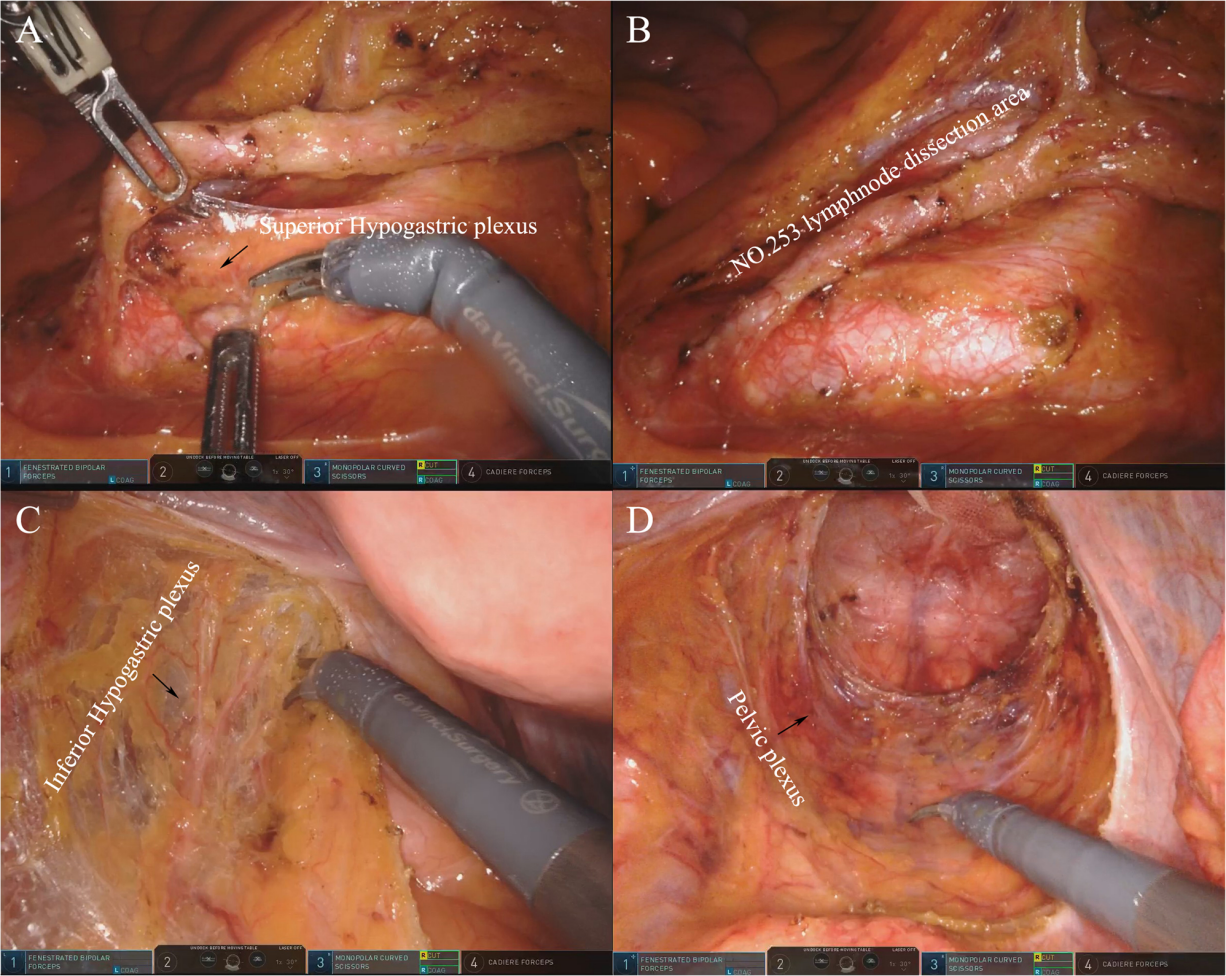
Neuroprotection in robotic TME surgery. **A**, **B** Protection of the inferior epigastric nerve during the group 253 lymph node dissection. **C** Protection of the inferior epigastric nerve during complete resection of the mesorectum. **D** Protection of the pelvic nerve during complete resection of the mesorectum.

Currently, combined with neoadjuvant chemoradiation, TME surgery is considered the standard therapeutic method for locally advanced rectal cancer [6]. However, even in the hands of expert surgeons, TME of lower rectal cancer still remains difficult, especially in patients with a narrow pelvis; male patients; obese patients, anteriorly located lesions and bulky tumors; or patients treated with neoadjuvant chemoradiotherapy [7, 8]. Moreover, even if the mesentery can be completely resected, protecting the superior epigastric nerve, inferior epigastric nerve, and pelvic nerve, which are crucial for maintaining urinary and sexual function, is also far from easy (Fig. 1). For these reasons, robotic approaches have gained significant recognition in TME of lower rectal cancers. However, using a robot in rectal cancer surgery is not without some challenges; the advantage it confers is also not as high as expected. Several studies have focused on comparing various aspects of robotic surgery to conventional laparoscopic surgery and found no statistically significant differences in perioperative complications, lymph nodes harvested, distal resection margin (DRM), pathological CRM, time of the first flatus, reoperation rate, local recurrence, and overall survival rate between these two groups in 3 years after operation. There is also no strong evidence proving the better surgical, functional, or oncological outcomes of robotic TME surgery [9•], especially compared with its applications in other surgical fields, such as urology and gynecology. In contrast, several studies have shown that robotic approaches require more operation time and cost, with statistical differences [10].

A longer operative time along with higher cost is widely considered the main disadvantage of robotic TME, when compared to the conventional laparoscopic procedure. The reason for the longer operative time is generally thought to be associated with the additional time taken to install the complicated robotic system. Furthermore, due to lack of haptic feedback and unskilled maneuvers, surgeons had to take more time to complete regular tasks under the robotic system. D’Annibale et al. [11] and Malak et al. [12] found that operative time significantly decreased as the number of cases accumulated in robotic surgeries. With regard to the learning curve, Sng et al. proved that senior colorectal surgeons can be familiar with basic robot skills such as docking and anatomy through 35 cases of robotic rectal cancer surgery. For patients with lower tumor location, it may be more challenging anatomically; hence, the curve is quite steep, up to about 120 cases [13]. Therefore, the experience and skill of surgeons might be important factors contributing to difference in operative time for robotic and laparoscopic TME. Some studies have also reported an even shorter operative time using a hybrid technique composed of a laparoscopic and robotic procedure [14]. In the author’s view, hybrid robotic surgery is only a technical transition to attain the full advantage of robotic surgery. It is believed that, with surgical teams gaining more experience in proper port placement and the standardization of every step, the robotic operating time can be further decreased.

The second main concern regarding robotic TME is the higher cost compared to that of conventional laparoscopic TME surgery. The reason for the high cost is mainly in the purchase and maintenance of equipment. This problem also has national and regional differences among various departments. It is believed that with the upgrade of robots in the future, this high cost will be effectively reduced.

Apart from disadvantages described above, the advantage of robotic rectal resection in avoiding damage to urinary and sexual functions has been demonstrated in several studies [15]. This advantage is mainly due to the better field of vision and flexible mechanical fingertips provided by robotic surgery. The wristed instruments of robotic approaches are small and highly flexible, with the ability to expose and separate tiny tissues, which dramatically reduces the damage to the pelvic nerves and blood vessels [16]. Therefore, it can provide better dissection of the avascular plane between the presacral fascia and fascia propria of the rectum, and preserve the integrity of the mesorectum without injuring peripheral tissues [17]. This unique advantage of the robotic system can provide a clear vision for recognition of tiny nerves, thus protecting urinary and sexual functions [17, 18]. One study showed that the incidence of partial or total erectile dysfunction was lower in robotic TME than that in conventional laparoscopic surgery [16]. A few other studies also reported the rate of postoperative erectile dysfunction; however, they failed to provide clear comparative data [19].

In addition, most published non-randomized controlled trial studies have confirmed that the clinical and pathological results are similar compared to laparoscopic surgery. These accumulated evidences prove the safety and feasibility of robotic TME. With development in technology and the proficiency of the operation team, operation time will be shortened and cost will be reduced. It is expected that more multicenter, prospective, randomized controlled trials aimed at evaluating safety, feasibility, economy, and long-term results will provide more key information on the research of robotic TME. A summary of the published experience of RTME in recent years is presented in Table 1.

**Table 1:** Summary of published experience of the robotic total mesorectal excision

Author, year	Baik et al. [20] (2009)	Patriti et al. [21] (2009)	Bianchi et al. [22] (2010)	Park et al. [23] (2010)	Baek et al. [24] (2011)	Kwak et al. [25] (2012)	Verheijen et al. [26] (2014)	Atallah et al. [27] (2015)	Kuo et al. [28] (2017)	Monsellato et al. [29] (2019)	Hu et al. [30] (2020)	Tan et al. [31] (2020)	Suhardja et al. [32] (2020)	Ye et al. [33] (2021)
Number of patients	56	29	25	41	41	59	1	4	15	3	20	1	1	13
Operation platform	da Vinci	da Vinci	da Vinci	da Vinci	da Vinci	da Vinci	da Vinci	da Vinci Si	da Vinci Si	da Vinci Si	da Vinci Xi	N/A	da Vinci Xi	da Vinci Si
Type of surgery	Robotic-assisted	Robotic-assisted	Totally robotic (75%)	Robotic-assisted	Robotic-assisted	Totally robotic	Robotic-assisted	Robotic-assisted	Totally robotic	Robotic-assisted	Robotic-assisted	Robotic-assisted	Totally robotic	Totally robotic (9); robotic-assisted (4)
Mean operating time (h)	190.1±45	202 ± 12	240 (170–420)	231.9±61.4	296 (150–520)	270 (241–325)	205	376 (140–409)	473 (335–569)	530 (440–600)	172.3±24.2	132	210	240 (195–270)
Mean blood loss (mL)	NA	NA	NA	NA	200 (20–2000)	NA	50	200 (50–300)	33 (30–50)	Inconsistent	82.0±107.1	20	160	60 (50–100)
Hospital stay (days)	5.7 ± 1.1	11.9 ± 7.5	6.5 (4–15)	9.9 ± 4.2	6.5 (2–33)	NA	3	4.3 (4-5)	12.2±1.5	10.6 (7–15)	8.8±4.2	6	5	7 (6–10)
Conversion rate (%)	0	0	0	0	7.3	0	0	0	13.3	0	0	0	0	0
Perioperative complications (%)	5.4	30.6	16	29.3	22	32.2	0	3 (75)	2 (13.3)	0	3 (75)	0	0	7 (6–10)
Mean number of LN harvested	18.4 ± 9.2	10.3 ± 4	10.3 ± 4	10.3 ± 4	13.1 (3–33)	20 (12–27)	NA	27 (15–39)	12 (8–18)	NA	18.7 ± 6.3	NA	24	15 (13–16)
Mean-free DRM (cm)	20 (12–27)	2.1 ± 0.9	NA	2.1 ± 1	3.6 (0.4–10)	2.2 (1.5–3.0)	NA	NA	1.4 (0.4–3.5)	NA	2.9 ± 1.3	NA	NA	2 (1.5–2.5)
Resection margin status (R0) (%)	92.9	100	100	95.1	97.6	98.3	100	100	100	100	3 (15)	NA	NA	100
Quality TME (I/II/III)	92.6/7.4/0	NA	NA	NA	NA	NA	100/0/0	100/0/0	100/0/0	100/0/0	90/10/0	NA	100/0/0	61.5/38.5/0

## Robotics in transanal TME (TaTME) surgery in locally advanced low rectal cancer

For middle–low rectal cancer, exposure of the operative field, rectal dissection, and transanal presacral ultra-low anastomosis can be challenging and increase the risk of anastomotic leakage. In 2010, Sylla et al. [34] first reported the TaTME technology, which has shown its unique technical advantages in overcoming these limitations, compared to conventional open and laparoscopic approaches. The anatomical basis of TaTME relies on a “bottom-up” dissection technique. This retrograde resection provides a magnified vision in line with pelvic structures. In this technique, it is easy to control the distal tumor margin, improving the identification and preservation of nerve tissue and making dissection safe and effective. Since the introduction of TaTME, it has aroused great interest in the CRC community. Several case series have suggested that TaTME is feasible and safe with regard to short-term outcomes and quality of the resected specimen, with a promising rate of CRM involvement lower than 6% [35]. However, despite its proven safety and feasibility, TaTME is still a challenging technique for most surgeons, with a longer learning curve [36, 37••]. Urethral injury is the most common and serious complication directly related to unfamiliarity with pelvic anatomy and less proficiency in surgical skills from the transanal phase [38]. Moreover, compared to robotic low anterior resection, higher involvement of the DRM has also been reported [39]. Therefore, it is difficult to maximize the advantages of laparoscopic-assisted TaTME surgery.

With the development in robotics, robotic TaTME (RTaTME) surgery has gradually come into the view of surgeons. Under the help of the da Vinci® Surgical System, RTaTME is still divided into transabdominal and transanal parts. The cooperation of the two-part operation has been described with different variations, either performed heterochronically or simultaneously in a double-team approach, according to the surgeon’s preference. Normally, the transabdominal part is completed with the assistance of robotic technology. Similarly, robotic-assisted abdominal part can be multi-port or single port, and under the assistance of robotics, the dissection of the transabdominal part may be more accurate. Recent reports have shown similar clinical and oncological results in comparing robotic and laparoscopic transabdominal surgical procedures. Therefore, no significant inferiority of robotic surgery compared to laparoscopic surgery seems to be detected at present, except in the conversion rates.

In addition to traditional abdominal robotic surgery, the transanal surgery team can also use the da Vinci System to perform surgery in the perineum. Traditionally, the transanal part can use transanal minimally invasive surgery (TAMIS), natural orifice specimen extraction surgery (NOSES), inter sphincter resection (ISR), or a reusable platform with rigid rectal scope TEM/TEO [40, 41] to complete bottom-up TME. The development of robotic technology enables the robot system to participate in the transanal part; some centers have completed this operation, but the oncological outcome is not ideal. Hu et al. [30] reported twenty cases of robotic TaTME. The total postoperative complication rate was 35% and the average length of the distal margin was (3.1 ± 1.3) cm. In all cases of this study, the distance from the tumor to the anal margin was 2–10 cm. However, in three cases, the circumferential margin was 1 mm, with cancer cells involved. These poor outcomes are related not only to the low location of the tumor, but also to the technical constraints, especially in the process of separating the mesorectum from the anal canal to the upward peritoneal fold and confirming the lower cutting edge.

**Fig. 2:**
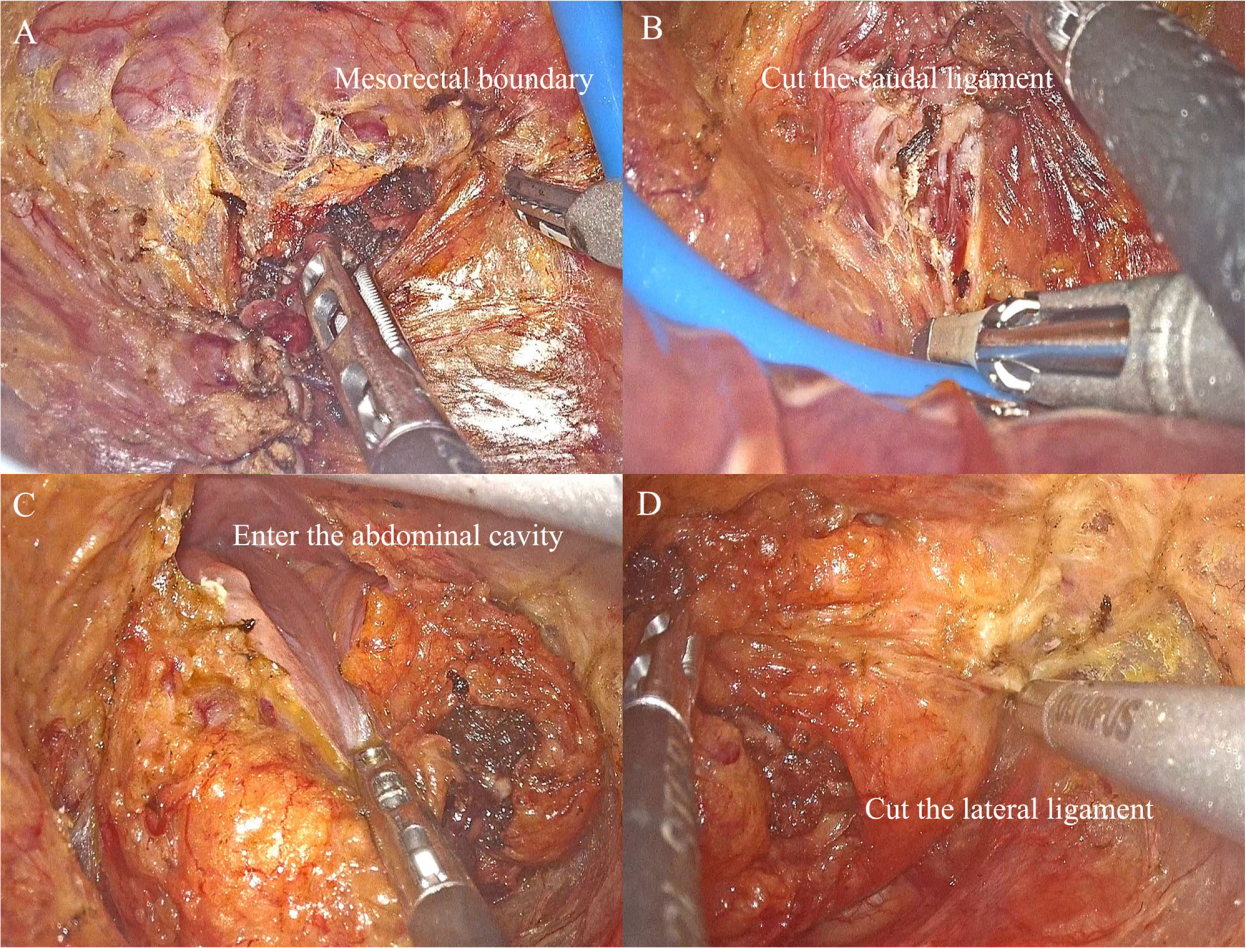
Laparoscopic transanal part of robotic TaTME surgery. **A** Find the boundary between the rectal mesorectum and the pelvic fascia from lateral direction. **B** Cut off the rectum caudate ligament from the rear. **C** Open the Denonvilliers’ fascia in the front and enter the abdominal cavity. **D** Cut off the lateral ligament.

Similar with conventional TaTME surgery, the development of RTaTME surgery is also inseparable from the progress of the transanal device; however, owing to the narrow operation field in the anal area, and the smaller-sized single-port transanal platform, external and internal arm conflicts make it difficult for a multi-arm robot to provide full display of its advantages in a single-port transanal surgery [42]. Therefore, most colorectal surgery centers still use the laparoscopic TAMIS platform to complete the transanal part. For maximum advantage of robot technology in the transanal part, designing a more flexible robot system for natural orifice surgery is the most important issue to be solved (Fig. 2). A summary of the published experience of RTaTME in recent years is presented in Table 2.

**Table 2:** Summary of published experience of RTaTME performed with the da Vinci® robotic platform

Author, year	Atallah et al. [43] (2013)	Atallah et al. [44] (2014)	Verheijen et al. [26] (2014)	Huscher et al. [45] (2015)	Gomez Ruiz et al. [46] (2015)	Kuo et al. [28] (2017)	Monsellato et al. [29] (2019)	Hu et al. [30] (2020)	Marks JH et al. [47] (2020)	Ye et al. [33] (2021)
Number of patients	1	3	1	7	5	15	3	20	2	13
Abdominal approach	Laparoscopic	Laparoscopic	Laparoscopic	Laparoscopic	Robotic	Single-port robotic + assistant port	Robotic (2), laparoscopic (1)	Robotic (2), laparoscopic (1)	Transabdominal single-incision laparoscopic (SILS)	Robotic (9), laparoscopic (4)
Transanal platform	GelPoint Path (daVinci® Si)	GelPoint Path (daVinci® Si)	GelPoint Path (daVinci® Si)	GelPoint Path (daVinci® Si)	GelPoint Path (daVinci® Si)	GelPoint Path (daVinci® Si)	GelPoint Path (daVinci® Si)	GelPoint Path (daVinci® Xi)	GelPoint Path (daVinci® SP)	GelPoint Path (daVinci® Xi)
Two-team approach	No	No	No	No	No	No	1/3	20/20	No	4/13
Mean operating time (min)	381	376	205	165.7 (85–220)	398 (270–450)	473 (335–569)	550 (440–600)	172.3 (135–215)	214.5 (72–357)	240 (195–270)
Mean blood loss (mL)	140	200	200	NA	90 (25–120)	33 (30–50)	NA	82 (30–500)	165(130–200)	60 (50–100)
Hospital stay (days)	No	4.3	3		6 (5–7)	12.2 (10–14)	10 (7–15)	8.8 (6–24)	3.5(3–4)	7 (6–10)
Conversion rate (%)	0	0	0	0	0	13.3	0	0	0	0
Hand-sewn anastomosis	0/1	2/3	0/1	0/7	2/5	15/15	3/3	2/20	2/2	8/13
Defunctioning stoma	Terminal ileostomy	Yes	Yes	Yes	Yes	5/15	Yes	14/18	Yes	Yes
Perioperative complications	No	Pulmonary embolism (1) Peristomal dermatitis/dehydration (1)	No	Anastomotic bleeding (1)	Anastomotic leak (1)	Mechanical bowel obstruction (1), wound infection (1)	Acute renal failure (1)	No	No	Duodenal hemorrhage (1) anastomotic leakage (1)
TME quality C/NC/I	0/1/0	1/2/0	1/0/0	6/1/0	5/0/0	15/0/0	3/0/0	18/2/0	2/0/0	8/5/0
CRM involvement	No	No	No	No	No	No	No	3/20	No	No
Distal margin involvement	No	No	No	No	No	No	No	No	No	No

Although RTaTME is still in its infancy, its potential in terms of mesorectal integrity, resection margins, number of lymph nodes harvested, and conversion rate is promising. With development of robotic systems, RTaTME should be the milestone progress step in the evolution of minimally invasive surgery.

## Robotics in lateral lymph node dissection in advanced rectal cancer

Increasing evidence has demonstrated that lateral lymph node metastasis (LLNM) is a major cause of systemic or local recurrence of advanced rectal cancer [48]. Patients with LLNM usually present with nearby organ involvement, resulting in a survival rate of less than 20% [49, 50]. LLND is effective in resecting metastatic lymph nodes and has been shown to decrease the local recurrence rate. LLND was first described in 1948 by Brunschwig, and its use for colorectal cancer was first described in 1959 by Butcher and Spjut [51]. In the early and mid-twentieth century, researchers from Europe, America, and Japan conducted a comprehensive study on the lateral lymph nodes of rectal cancer and confirmed that LLNM mainly occurs in patients with late low rectal cancer. European and American scholars generally believe that LLNM should be classified as systemic metastasis. If the lateral lymph nodes (except the internal iliac lymph nodes) are metastatic, it is defined as distant metastasis. From the 1950s to the 1980s, European and American researchers believed that LLND usually had complications, such as long operation time and increased intraoperative bleeding, affecting micturition and sexual function, and not being able to improve the survival rate of patients [52]. Therefore, with the wide application of neoadjuvant therapy in rectal cancer in the 1990s, most European and American researchers replaced LLND with neoadjuvant + TME. However, LLNM and local recurrence still occur despite neoadjuvant treatment with TME. The time Japanese scholars took to perform LLND is basically the same as that in Europe and the USA, but the difference is that Japanese researchers generally believe that lateral lymph nodes are regional lymph nodes of rectal cancer. In Japan, TME with LLND is the standard surgical treatment for stage II/III advanced low rectal cancer. Before surgery, a proper evaluation of the LLN status should be performed. Japanese scholars are committed to continuously adjusting surgical indications and continuously improving surgical methods to significantly reduce the occurrence of postoperative complications. With the help of the laparoscopic approach, the complication and mortality rates of LLND have significantly decreased, and the 5-year overall survival rates have increased from <5 [53] to 22–66% [54]. Laparoscopic LLND in TME was performed relatively late in China, mainly in patients with low rectal cancer suspected of LLNM in imaging [54].

However, the laparoscopic approach still currently encounters technical difficulties in preserving the pelvic plexus in LLND surgery. Therefore, more sophisticated robotic technology should be developed. Enhanced 3D visualization and magnification can improve the surgeon’s depth of perception and clarity of vision in the pelvis, to discern the smallest nerve fibers. Robotic Endo Wrists® can also provide significantly more flexible articulation beyond the limits of human wrist movements to facilitate superior operative dexterity with augmented precision for surgical dissection while preserving stable tissue resection.

**Fig. 3.**
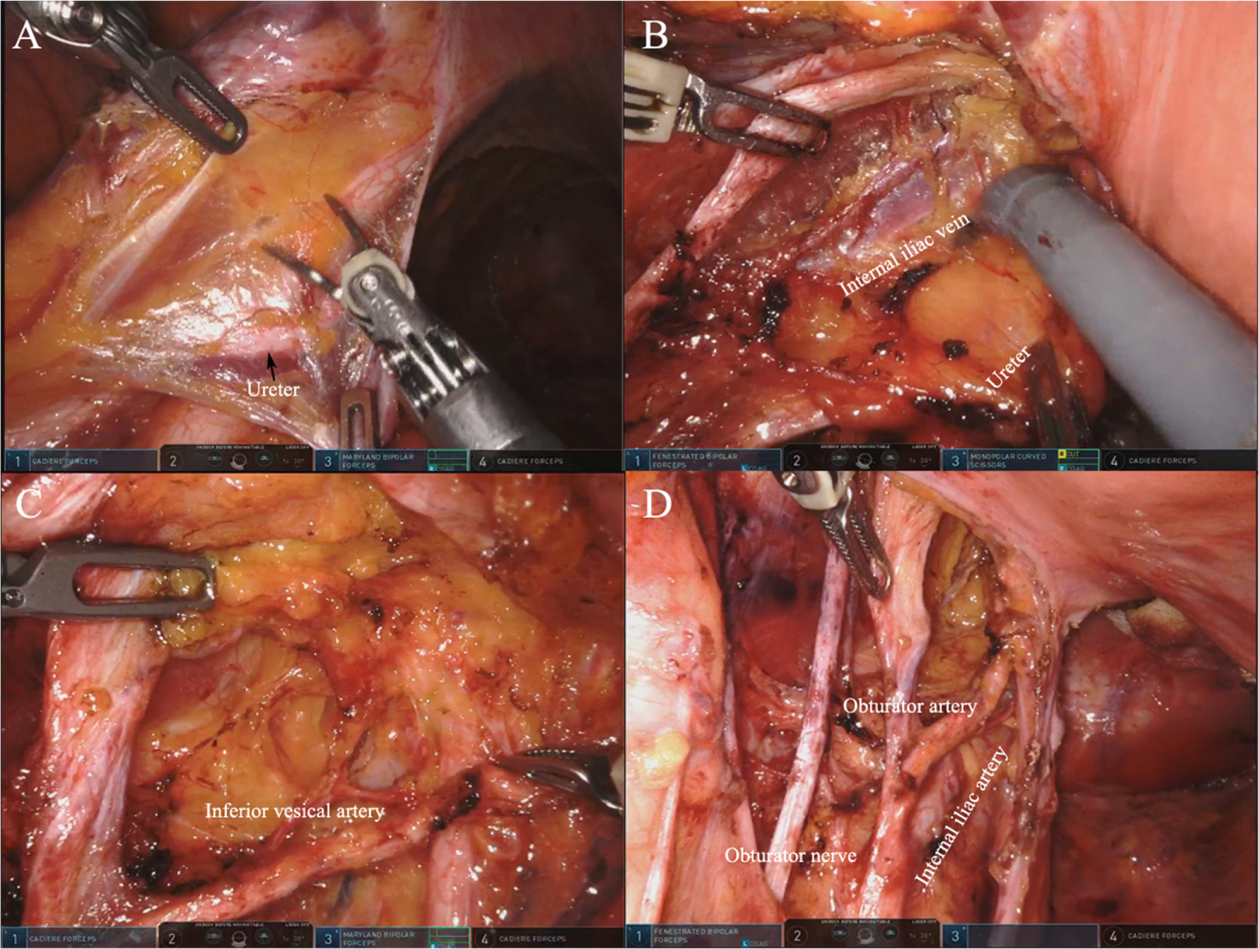
Robotic system–assisted left pelvic exenteration. **A** Open the peritoneum on the outside of the ureter. **B** Expose the internal iliac artery and vein, and separate the urinary fascia plane composed of the pelvic plexus and ureter. **C** Continue to separate distally along the internal iliac artery, clean the lymph nodes, and identify the inferior bladder artery. **D** Display the lateral region after complete lymph node clearance.

The first robotic-assisted LLND for locally advanced rectal cancer was described by Nanayakkara et al. in 2014 [55] using the da Vinci® Surgical System. Since then, there has been a steady increase in the number of case reports demonstrating its safety and feasibility in multivisceral resections for locally advanced and recurrent rectal cancers worldwide (Table 3). Available evidence shows that robotic platforms require longer operative time and are more costly than conventional laparoscopic approaches. Factors that increase the operating time of robotic-assisted approaches include large tumors, edema due to preoperative treatment, and intraoperative bleeding. However, in terms of its security, robotic-assisted pelvic exenteration has certain advantages in preserving the pelvic nerves and branch vessels (Fig. 3).

**Table 3:** Original studies utilizing the da Vinci® Surgical System for multivisceral pelvic exenteration surgery for locally advanced including recurrent rectal cancers

Author, year	Number of patients	Operation platform	Type of surgery	Mean operating time (h)	Mean blood loss (mL)	Perioperative complications	Mean ITU stay (days)	Hospital stay (days)	Conversion rate (%)	Perioperative complications	Resection margin status (R0)	Recurrence rates
Shin et al., [56] (2014)	3	da Vinci	Robotic-assisted	8.9 (8–9.7)	530 (300–700)	Vesico-urethral anastomotic leak (1)	NA	18 (8–28)	NO	NO	18 (8–28)	18 (8–28)
Nanayakkara et al. [55] (2014)	1	da Vinci	Robotic-assisted	NA	NA	NO	NA	8	NO	NO	1/1 (100%)	NA
Winters et al. [57] (2015)	3	GelPoint Path (daVinci® Si)	Robotic	10.1 (9.5–11)	550 (350–800)	NO	1	7	NO	NO	1/3	NA
Shin et al., [58] (2016)	22	da Vinci	Robotic	7 (5.5–8.5)	417.5 (337–496)	12/22 (52%) Pelvic abscess (4); hemorrhage (1); urinary retention (3); urinary leak (1); ileus (5); re-admissions (6); re-operations (3)	NA	4 (3–5.5)	NO	NO	22/22 (100%)	NA
Raj Kumar et al. [59] (2020)	1	GelPoint Path (da Vinci® Si)	Robotic	9	750	NO	NA	NA	NO	NO	1/1 (100%)	Disease free at 6 months
Heah et al. [60] (2020)	3	GelPoint Path (daVinci® S)	Robotic-assisted	NA	700 (600–800)	NO	NA	12.6	NO	NO	2/3 (67%)	NA
Smith et al., [61] (2020)	8	GelPoint Path (da Vinci® Si=5; Xi=3)	Robotic	8.3 (6–10)	Received 2 units (2)	NO	1 (0–3)	15 (7 to 26)	NO	NO	8/8 (100%)	Disease free at 12 months
Williams et al. [62] (2021)	5	GelPoint Path (da Vinci® Si/Xi/S)	Robotic	7.8 (3–11)	520 (150–1000)	Mortality (1)	1 (1–1)	9 (6–34)	NO	NO	4/5 (80%)	3/5 at 21 and 24 months

## Artificial intelligence and robotic surgery: future direction

AI is the field of computer science devoted to building smart machines capable of performing tasks that typically require human-level intelligence [63]. The development of AI algorithms has permeated the medical field with great success, such as its wide application in the diagnosis and treatment of a variety of cancers, especially colorectal cancer, which is now attracting substantial attention.

In general, AI applications in the medical field have two main branches: virtual and physical. The virtual branch of AI comprises machine learning (ML) and deep learning (a subset of ML) [64]. The physical branch of AI includes medical devices and robots, such as the da Vinci Surgical System and nanorobots for targeted drug delivery [65]. Conventional mechanical robots transmit the surgeon’s hand movements to the surgical target through the tremor-filtered movements of surgical instruments. With the import of AI, the next iteration of surgical robots conforms human-initiated actions to a personalized surgical plan by leveraging 3D digital segmentation generated prior to surgery. The addition of AI to robotic surgery ensures the precise implementation of preplanned steps of operative surgery, to avoid harm by decreasing the deviation and thus achieving improved patient outcomes.

In addition to these basic functions, intelligent robotic surgery is fast approaching to an era in which a robot can either perform preprogrammed tasks or learn from its own experience through a feedback pipeline of good and not-so-good outcomes (reinforcement learning) [66]. An intelligent robot will recognize tissues or organs and perform precise execution supervised by a surgeon, or automatically. To achieve this goal, intelligent robot established artificial neural networks (ANNs) to drive the designed deep learning models (DLMs). ANNs are digital simulants of biological nervous systems. Through ANNs, DLMs would not only discern the blood vessels in relation to a tumor clearly but also provide incisive views on how an expert surgeon would negotiate tricky bends in a troubled situation. Moreover, intelligent robots are capable of selecting appropriate instruments and providing high-quality support in the decision-making process of the surgeon. Meanwhile, the application of AI can also have use in presurgical planning and postoperative surgical skill evaluation using big data capture and logistics data sharing technology in robotic surgery. With the accumulation of more data, the research and development of autonomous robots is also ongoing. In 2015, with the collaboration between Google and Johnson, Verb Surgical successfully introduced robotic AI to start a new era of robotic-guided, rather than robot-assisted, surgery [66].

Although AI technology has broad prospects, there are still significant challenges and pitfalls. The most important problem in the future of AI in medicine is ensuring data privacy and confidentiality. The new model of health data ownership with individual rights, high security platform, and potential government intervention will provide a reasonable solution to this problem in the future. It is believed that developing a surgery technology that is fully performed by robots is no longer distant.

## Conclusions

This paper reviews the application of robot technologies in several mainstream radical resections of rectal cancers from the perspective of surgery. Although robots have brought new technological innovations to rectal cancer surgery, they also face many difficulties and challenges. The development of science and technology will accelerate the integration of advanced robot platforms and artificial intelligence. The limitation of this paper is that the number of patients involved in the literature on robotic surgery is relatively small, as well as lacks the randomized research and prospective evaluation of the technology. What is worth looking forward to are several ongoing clinical research investigating robotic surgery on low rectal cancer have been enrolling a larger number of cases and will bring us more convinced results. For example, a prospective randomized controlled trial from Hongkong (ClinicalTrials.gov Identifier: NCT04091620) is comparing the outcomes of transanal total mesorectal excision versus robotic total mesorectal excision for mid- and low rectal cancer in aimed 103 patients. Another matched parallel cohort trial from France (ClinicalTrials.gov Identifier: NCT03574493) compared the outcomes of high surgical risk mid- to low rectal cancer patients under laparotomy vs laparoscopy vs robotic vs TaTME rectal surgery is aiming to enroll 1300 participants and lasts 6 years. A prospective observational cohort trial in Spain (ClinicalTrials.gov Identifier: NCT04936581) aims to enroll 200 patients to compare the outcomes under open approach versus laparoscopic approach versus robotic approach in total mesorectal excision. With the increase of surgical cases, more and more multicentered and prospective research will provide a more significant reference for evaluating the safety, effectiveness, surgical, and short-term oncological effects of robotics for rectal cancer surgery.
